# Clinical care guidance in patients with diabetes and metabolic dysfunction–associated steatotic liver disease: A joint consensus

**DOI:** 10.1097/HC9.0000000000000571

**Published:** 2024-10-30

**Authors:** Jee-Fu Huang, Tien-Jyun Chang, Ming-Lun Yeh, Feng-Chih Shen, Chi-Ming Tai, Jung-Fu Chen, Yi-Hsiang Huang, Chih-Yao Hsu, Pin-Nan Cheng, Ching-Ling Lin, Chao-Hung Hung, Ching-Chu Chen, Mei-Hsuan Lee, Chun-Chuan Lee, Chih-Wen Lin, Sung-Chen Liu, Hwai-I Yang, Rong-Nan Chien, Chin-Sung Kuo, Cheng-Yuan Peng, Ming-Ling Chang, Chung-Feng Huang, Yi-Sun Yang, Hung-Chih Yang, Han-Chieh Lin, Horng-Yih Ou, Chun-Jen Liu, Chin-Hsiao Tseng, Jia-Horng Kao, Wan-Long Chuang, Chien-Ning Huang, Pei-Jer Chen, Chih-Yuan Wang, Ming-Lung Yu

**Affiliations:** 1 Hepatobiliary Division, Department of Internal Medicine, Kaohsiung Medical University Hospital, Kaohsiung Medical University, Kaohsiung, Taiwan; 2 Graduate Institute of Clinical Medicine, Kaohsiung Medical University, Kaohsiung, Taiwan; 3 College of Medicine and Center for Liquid Biopsy and Cohort Research, Kaohsiung Medical University, Kaohsiung, Taiwan; 4 Department of Internal Medicine, National Taiwan University Hospital, Taipei, Taiwan; 5 Division of Endocrinology and Metabolism, Department of Internal Medicine, Kaohsiung Chang Gung Memorial Hospital, Kaohsiung, Taiwan; 6 School of Medicine, College of Medicine, Chang Gung University, Taoyuan, Taiwan; 7 Division of Gastroenterology and Hepatology, Department of Internal Medicine, E-Da Hospital, School of Medicine, I-Shou University, Kaohsiung, Taiwan; 8 Institute of Clinical Medicine, National Yang Ming Chiao Tung University Faculty of Medicine, Taipei, Taiwan; 9 Healthcare and Services Center and Therapeutic and Research Center of Liver Cancer, Taipei Veterans General Hospital, Taipei, Taiwan; 10 Division of Endocrinology and Metabolism, Department of Internal Medicine, Taipei City Hospital Renai Branch, Taipei, Taiwan; 11 Department of Internal Medicine, National Cheng Kung University Hospital, Tainan, Taiwan; 12 College of Medicine, National Cheng Kung University, Tainan, Taiwan; 13 Department of Internal Medicine, Cathay General Hospital, Taipei, Taiwan; 14 Division of Hepatogastroenterology, Department of Internal Medicine, Kaohsiung Chang Gung Memorial Hospital, Kaohsiung, Taiwan; 15 Division of Endocrinology and Metabolism, Department of Medicine, China Medical University Hospital, Taichung, Taiwan; 16 School of Chinese Medicine, China Medical University, Taichung, Taiwan; 17 Division of Endocrinology and Metabolism, Department of Internal Medicine, MacKay Memorial Hospital, Taipei, Taiwan; 18 Department of Medicine, Mackay Medical College, Taipei, Taiwan; 19 Genomics Research Center, Academia Sinica, Taipei, Taiwan; 20 Department of Gastroenterology and Hepatobiliary Disease, Linkou Chang Gung Memorial Hospital, Taipei, Taiwan; 21 Division of Endocrinology and Metabolism, Department of Medicine, Taipei Veterans General Hospital, Taipei, Taiwan; 22 School of Medicine, National Yang Ming Chiao Tung University, Taipei, Taiwan; 23 Department of Internal Medicine, Center for Digestive Medicine, China Medical University Hospital, Taichung, Taiwan; 24 School of Medicine, China Medical University, Taichung, Taiwan; 25 Division of Endocrinology and Metabolism, Department of Internal Medicine, Chung-Shan Medical University Hospital, Taichung, Taiwan; 26 Department and Graduate Institute of Microbiology, National Taiwan University College of Medicine, Taipei, Taiwan; 27 Division of Gastroenterology and Hepatology, Taipei Veterans General Hospital, Taipei, Taiwan; 28 Division of Gastroenterology & Hepatology, Hepatitis Research Center, National Taiwan University Hospital, Taipei, Taiwan; 29 School of Medicine and Doctoral Program of Clinical and Experimental Medicine, College of Medicine, Center of Excellence for Metabolic Associated Fatty Liver Disease, National Sun Yat-sen University, Kaohsiung, Taiwan

**Keywords:** care guidance, insulin resistance, liver fibrosis, metabolic dysfunction–associated steatotic liver disease, type 2 diabetes mellitus

## Abstract

Metabolic dysfunction–associated steatotic liver disease (MASLD) is the most prevalent chronic liver disease worldwide, affecting >30% of the global population. Metabolic dysregulation, particularly insulin resistance and its subsequent manifestation as type 2 diabetes mellitus, serves as the fundamental pathogenesis of metabolic liver disease. Clinical evidence of the recent nomenclature evolution is accumulating. The interaction and impacts are bidirectional between MASLD and diabetes in terms of disease course, risk, and prognosis. Therefore, there is an urgent need to highlight the multifaceted links between MASLD and diabetes for both hepatologists and diabetologists. The surveillance strategy, risk stratification of management, and current therapeutic achievements of metabolic liver disease remain the major pillars in a clinical care setting. Therefore, the Taiwan Association for the Study of the Liver (TASL), Taiwanese Association of Diabetes Educators, and Diabetes Association of the Republic of China (Taiwan) collaboratively completed the first guidance in patients with diabetes and MASLD, which provides practical recommendations for patient care.

## INTRODUCTION

### Construction process of the guidance

Fatty liver disease, also known as steatotic liver disease (SLD), is the most common liver disorder globally.^1^ It comprises a crucial component of metabolic dysfunction–associated steatotic liver disease (MASLD), formerly named. It may progress to cirrhosis and HCC.^2^,^3^ The interaction and impacts are bidirectional between MASLD and type 2 diabetes mellitus. Hepatic steatosis driven by either de novo lipogenesis or adipose tissue dysfunction significantly increases the risk of developing diabetes.^4^ Conversely, the prevalence of MASLD in diabetes patients is 65%, twice that of the general population, with a prevalence of significant fibrosis approaching 20%.^4^,^5^ Furthermore, diabetes exerts detrimental effects not only on the liver but also on extrahepatic systems in patients with MASLD, as evidenced by the increased risk of cardiovascular disease (CVD), stroke, chronic kidney disease (CKD), and all-cause mortality.^6^ Patients with diabetic MASLD are at higher risk of cancer and CVD mortality compared to nondiabetic counterparts. Taken together, from the perspective of holistic health care, the co-occurrence of diabetes poses notable adverse effects on individuals with MASLD, necessitating timely and effective management.

Taiwan is no exception to the high prevalence of both diabetes and MASLD. Hence, there is a pressing need to develop guidance for clinicians in terms of patient care and disease management. Currently, solid guidance has rarely been developed for clinical management in this issue. The debut of the guidance will not only be beneficial for the management of Taiwanese patients but also be the excellent reference for other regions where both MASLD and diabetes are prevalent. The concept of the guidance was initiated by leaders of the Taiwan Association for the Study of the Liver (TASL), Taiwanese Association of Diabetes Educators, and Diabetes Association of Taiwan in 2022. The first consensus meeting inviting experts from the societies was held in early 2023. Progress was made in several consensus meetings that year in terms of knowledge updating, literature review, statement structure design, and timelines, etc. Finally, a total of 18 position statements have been proposed (Table 1). Each position statement was made only when the consensus agreement was reached after scientific evidence review and discussion. The guidance released from the results of the consensus meetings will hopefully provide the appropriate management guidance for primary care providers.

**TABLE 1:** Position statements 1–18 for patients with diabetes and MASLD

PS 1	A multidisciplinary patient-centered approach is mandatory in patients with MASLD and diabetes.
PS 2	MASLD encompasses patients exhibiting steatosis and metabolic abnormalities.
PS 3	The presence of concurrent diabetes in patients with MASLD could lead to hepatic and systemic adverse effects.
PS 4	Insulin resistance plays a central role in the development of MASLD and diabetes. It is associated metabolic dysregulation highlights the importance of managing insulin resistance–associated diseases.
PS 5	The projected increase in MASLD prevalence in the coming decades underscores the significance of primary prevention. Vital strategies, including body weight management and glycemic control, play a crucial role in effective MASLD prevention.
PS 6	About 15%–20% of Asia-Pacific subjects with MASLD are lean. The risk factors, clinical presentation, and outcomes of those with lean MASLD are similar to those in obese or overweight populations.
PS 7	MASLD is associated with an increased incidence and prevalence of diabetes.
PS 8	Patients with diabetes face an increased risk of advanced fibrosis. Screening for fibrosis is recommended.
PS 9	Determination of liver disease severity is needed upon diagnosis of MASLD.
PS 10	Screening strategies for diabetes in MASLD include fasting plasma glucose level, hemoglobulin A1c level, or oral glucose tolerance test.
PS 11	Patients with diabetes or MASLD should be evaluated for their risk of atherosclerotic cardiovascular disease.
PS 12	Liver fibrosis assessment is essential for every patient with MASLD. Noninvasive assessment is recommended. FIB-4 is preferred.
PS 13	For MASLD and diabetes patients with FIB-4 >2.67, referral to a hepatologist is recommended due to the higher risk of liver-related events.
PS 14	MASLD is associated with an increased risk of chronic kidney disease and diabetic neuropathies.
PS 15	Lifestyle interventions to achieve a minimum of 7% body weight reduction and maintain 150 min/wk of moderate-intensity physical activity are highly effective in preventing incident diabetes and reducing progression of MASLD.
PS 16	Adherence to the healthy diet patterns such as Mediterranean diet and Japanese diet is beneficial to diabetes control and to reduction of steatosis and fibrosis.
PS 17	Some weight reduction agents and classes of antidiabetes drugs such as incretin-mimetics and pioglitazone show promising therapeutic potentials. The dual GIP/GLP-1 receptor co-agonist could lead to 20% of weight reduction in addition to significant steatosis reduction.
PS 18	For patients with diabetes and MASLD, adequate glycemic control is recommended to reduce HCC occurrence.

Abbreviations: FIB-4, fibrosis-4; GIP/GLP-1, glucose-dependent insulinotropic peptide/glucagon-like peptide 1; MASLD, metabolic dysfunction–associated steatotic liver disease; PS, position statement.

### The path of nomenclature changes

In 1980, Ludwig et al^7^ first coined NAFLD, which refers to the development of steatosis without significant alcohol consumption while sharing many histopathological features with alcohol-associated liver disease. Systemic and hepatic insulin resistance (IR) is an essential component of the pathogenesis of MASLD, which was first described by Marchesini et al^8^ in 1999. This characterization of MASLD overemphasizes the lack of significant alcohol consumption while underemphasizing the role of metabolic risk factors. In 2020, an international consensus group developed a new nomenclature of metabolic dysfunction–associated fatty liver disease (MAFLD) to address these issues.^9^,^10^ In 2023, the American Association for the Study of Liver Diseases and the European Association for the Study of the Liver endorsed the new nomenclature of SLD based on an affirmative and nonstigmatizing approach.^3^ SLD was chosen as an overarching term to encompass the various etiologies of steatosis. NAFLD was renamed MASLD, encompassing patients who have hepatic steatosis and at least 1 cardiometabolic risk factor. All of the abovementioned efforts to precisely define metabolic liver disease indicate not only the clinical importance but also the heterogenicity of this complex metabolic disease.

### Bidirectional relationship between diabetes and MASLD

There is accumulating evidence indicating that the presence of diabetes represents a significant risk factor in MASLD and should be treated with utmost seriousness. Diabetes significantly increases fibrosis progression, and fibrosis predicts long-term outcomes, including liver-related complications and all-cause mortality in MASLD.^11^,^12^ In addition, diabetes promotes the development of HCC. The cumulative burden of adverse metabolic effects correlates with an elevated risk of cirrhosis and HCC in a dose-dependent manner. Moreover, diabetes has emerged as the sole independent metabolic risk factor for HCC, underscoring its critical role in MASLD progression.^13^

On the other side, MASLD negatively affects the incidence and outcomes of diabetes. MASLD is associated with a 2- to 5-fold increased risk of developing diabetes, independent of age, sex, adiposity, and other metabolic risk factors.^14^ This risk is further elevated in patients with hepatic steatosis and/or advanced fibrosis. The risk of developing diabetes may also be observed among males, smokers, physically inactive individuals, and those with a body mass index (BMI) ≥25 kg/m².^15^

### Crucial role of IR in the development of diabetes and MASLD

IR is a complex condition characterized by the reduced responsiveness of peripheral target tissues to the actions of insulin. It leads to the impaired regulation of blood glucose and abnormal lipid metabolism and is often accompanied by elevated insulin levels to compensate for the reduced sensitivity.^16^ IR is characterized by insulin-mediated blood glucose management disorders, blood glucose utilization disorders, abnormal lipid accumulation, and increased lipid decomposition activities in adipocytes. It is associated with various metabolic disorders, including obesity, diabetes, CVD, and other metabolic disorders.^17^ Obesity, particularly the accumulation of intra-abdominal and intrahepatic fat, is strongly associated with both IR and diabetes. Furthermore, IR is independently linked to chronic macrovascular and microvascular complications associated with diabetes. These complications include CVD and extrahepatic diseases, including atherosclerotic cardiovascular disease (ASCVD) and extrahepatic cancers.^18^ position statements 1–4 in Table 1.

## EPIDEMIOLOGY AND THE BIDIRECTIONAL INTERACTION

### Diabetes and MASLD: Globally and in Taiwan

The global diabetes patient numbers will soar to 643 million by 2030, and to 783 million by 2045. Geographically, in 2021, >200 million patients with diabetes lived in the Western Pacific Region, which accounted for more than one-third (38%) of the total number of adults living with diabetes. The number of patients with diabetes in this region will increase by 27%, reaching 260 million by 2045, and the prevalence of diabetes will increase by 21% to reach 14.4% in 2045.^19^ Taiwan has the third highest prevalence of diabetes (11.9%) globally at the state level. There were 2.45 million adult patients with diabetes in Taiwan in 2021, with a prevalence of 13.1%.^20^

There is a strong correlation between the prevalence of MASLD and overweight and obesity. The global prevalence of MASLD is 39.2%, with the highest prevalence in Europe and Asia.^21^ Among overweight or obese adults, the overall prevalence of MASLD is 50.7%.^22^ The prevalence of MASLD has reached 18% among individuals with lean or normal weight in the Asian population.^23^

Large-scale epidemiological studies in Taiwan have demonstrated that the prevalence of MASLD ranges from 44.5% to 58.6%.^24^ A recent modeling study using the pooled database estimated the prevalence of MASLD in 2019 to be 21.8%, with a projected increase to 23.2% by 2030.^25^

### Risk factors for diabetes and MASLD

Obesity is the leading modifiable risk factor for diabetes, particularly for those with a genetic predisposition.^26^,^27^ Overnutrition and lack of exercise not only contribute to obesity but also increase the risk of diabetes.^28^ Certain medical disorders such as prediabetes, gestational diabetes, and polycystic ovary syndrome may increase the risk of diabetes.

The risk factors for diabetes could potentially also be risk factors for MASLD. The risk factors for MASLD include high BMI, diabetes, and metabolic alterations. In addition, increasing age, unhealthy diet, and lack of physical activity may contribute to obesity and IR and further increase the risk of MASLD. Furthermore, genetic predispositions (eg, genetic variation of the patatin-like phospholipase domain-containing protein and transmembrane 6 superfamily member 2 genes) are linked to a higher risk of MASLD.^28^,^29^

### Impact of diabetes on MASLD

Diabetes is a significant risk factor for advanced fibrosis and cirrhosis in patients with obesity and MASLD.^30^ Diabetes is also a significant risk factor in patients with alcohol-associated liver disease (ALD)-MASLD for the development of liver fibrosis and accelerated fibrosis progression.^31^ Diabetes increases the risk of HCC development in the general population, even in patients without cirrhosis.^32^ Interestingly, adequate glycemic control is associated with a lower risk of HCC in patients with diabetes with MASLD.^33^ The risk of HCC associated with diabetes appears to be highest in MASLD and chronic hepatitis C (CHC) infection, followed by ALD and chronic hepatitis B infection.^32^ Although successful antiviral therapy by direct-acting antivirals greatly reduces HCC risk in patients with CHC, diabetes is still an independent risk factor of de novo HCC among those with virus eradication.^34^ Moreover, a recent study showed that metformin greatly reduces HCC risk after successful antiviral therapy in patients with CHC with diabetes.^35^

### Impact of MASLD on diabetes

Patients with MASLD have a 2.2-fold increased risk of incident diabetes.^14^ This risk remains significant even after adjustment for age, sex, adiposity measures, family history of diabetes, fasting glycemia, dyslipidemia, hypertension, smoking, and physical activity.

Notably, lean patients with MASLD have a higher risk of diabetes than patients with obesity or who are overweight without MASLD, even if they are nonobese without diabetes at baseline.^36^,^37^ Furthermore, the risk of incident diabetes parallels the fibrosis in MASLD determined by biopsy or clinical scores such as NAFLD fibrosis score and fibrosis-4 (FIB-4).^37^,^38^ On the other hand, improvement or resolution of MASLD, irrespective of changes in body weight, reduces the risk of incident diabetes.^39^ In individuals whose diabetes relapses after initial remission, accumulation of hepatic and pancreatic fat precedes the recurrence of diabetes.^40^ In addition, patients with both type 1 and type 2 diabetes with MASLD increase insulin requirements to achieve comparable glycemic control, indicating that diabetic patients with MASLD need more intensive diabetes treatment.^41^

### Lean MASLD and diabetes

The global prevalence of MASLD in lean individuals ranges from 5% to 26%, and lean MASLD constitutes 15%–50% of all MASLD cases.^42^,^43^,^44^ A high percentage (15%–21%) of Asia-Pacific MASLD subjects are lean, and the proportion of nonobesity can be as high as 75% in Indians with MASLD.^45^ The proportion of nonobesity is 45% in Taiwanese adolescents with MASLD.^46^ Epidemiologic data regarding the prevalence of MASLD in the lean population remain unclear.^44^ For a diagnosis of either lean MASLD or MASLD, 2 components are essential: the presence of hepatic steatosis and clinical evidence of metabolic dysregulation. The risk factors, clinical presentation, and outcomes of those with lean MASLD are similar to those in obese or overweight populations. Key factors contributing to the development of MASLD or MAFLD in lean populations include lifestyle, environmental, and ethnic factors.^46^,^47^ position statements 5–7 in Table 1.

## DIAGNOSIS AND SCREENING

### Relevant patient history and laboratory tests

The diagnosis of MASLD is established by the presence of metabolic dysfunction, leading to both hepatic and extrahepatic manifestations. MASLD constitutes a diverse spectrum of metabolic liver diseases. At present, there are no universally accepted screening protocols in place. Consequently, the initial step in developing an effective screening strategy is to identify the at-risk population.^10^ Patient history and laboratory data can help identify patients at high risk and are critical for MASLD screening and prevention. History taking should be conducted to find evidence of metabolic dysfunction and chronic liver diseases. Individuals with a metabolic medical history are eligible for MASLD screening. Therefore, a comprehensive assessment of the patient’s liver disease history, such as ALD, chronic hepatitis B, or CHC, is essential. The initial laboratory tests to screen for MASLD should focus on liver function tests and metabolic alterations. Additionally, platelet count is important for assessing the stage of liver fibrosis. Concomitant imaging study could be performed for those who are at risk for MASLD.

### Screening strategies for diabetes and MASLD

The changes in glucose homeostasis, lipid metabolism, and insulin sensitivity are common alterations characterized in both MASLD and diabetes. High IR is the predictive factor significantly associated with MASLD, even in patients with normal BMI.^24^ Therefore, patients with MASLD should be screened for diabetes.^48^ Recently, several advocacy efforts were addressed for an early diagnosis of MASLD and for screening all patients with diabetes, with multidisciplinary works for a more proactive approach.^49^,^50^ In patients with diabetes, the presence of MASLD should be identified irrespective of transaminase levels, given their high risk of fibrosis. Age (above 50 y), IR, and features of metabolic syndrome all increase the probability of NASH with a more severe fibrosis stage and cirrhosis.^51^

### Noninvasive tests and imaging studies

Numerous noninvasive test scores, such as the SteatoTest, fatty liver index, hepatic steatosis index, lipid accumulation product, and index of MASLD liver fat score, have been proposed for steatosis detection. While some have been validated independently, comparing their diagnostic performance is challenging.

Ultrasound-based methods offer moderate diagnostic accuracy in assessing hepatic steatosis. Conventional B-mode ultrasonography is readily available, cost-effective, and suitable for initial screening. The controlled attenuation parameter is a well-validated ultrasound-based quantitative measurement of liver fat. However, MRI-derived proton density fat fraction stands out with the highest level of diagnostic accuracy but with high cost and limited availability.

All patients with MASLD should be screened and assessed for liver fibrosis since advanced fibrosis is associated with disease progression, risk of HCC, variceal bleeding, and long-term outcomes.^52^ The current gold standard for assessing fibrosis is liver biopsy; however, it is invasive, inconvenient, and has limitations for continuous monitoring.^53^ Noninvasive methods for diagnosing fibrosis have been greatly established and performed as well as biopsy-assessed fibrosis in predicting long-term outcomes in patients with MASLD.^52^ Image-based methods included vibration-controlled transient elastography or liver stiffness measurement, shear wave elastography and MRI-based elastography.^54^ The serological markers included FIB-4 index, AST/ALT ratio, AST-to-platelet ratio index, nonalcoholic fatty liver disease fibrosis score, enhanced liver fibrosis test, and FibroMeter MASLD.^54^,^55^ The FIB-4 index is currently considered practical for general practitioners since it is readily available, simple, and easily calculated. The method includes age factor, and the cutoffs should be tailored to age to improve the accuracy.^56^

### When to perform the liver biopsy

**FIGURE 1:**
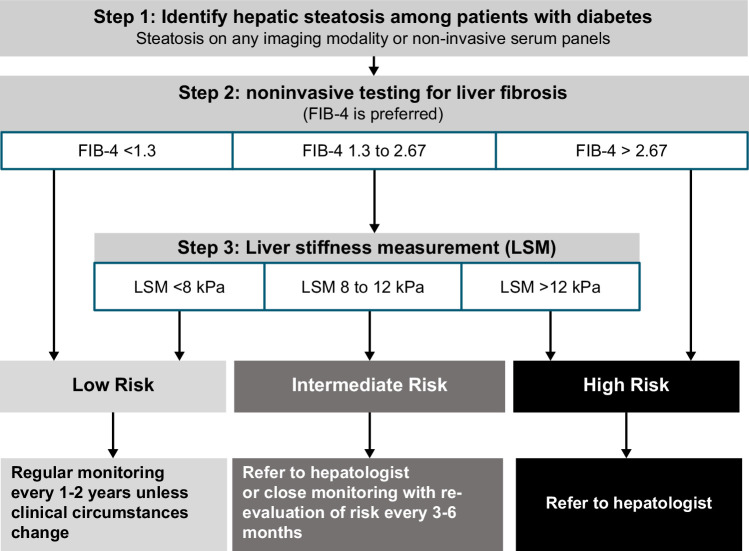
The screening and fibrosis assessment algorithm in metabolic dysfunction–associated steatotic liver disease. Abbreviations: DM, diabetes; FIB-4, fibrosis-4; LSM, Liver stiffness measurement.

A two-tier algorithm using the FIB-4 index (<1.3, 1.3–2.67, >2.67) as the first-line test, followed by FibroScan (<8, 8–12, >12 kPa), can be implemented sequentially to stratify the risk of liver fibrosis in patients with MASLD.^57^,^58^ Patients with FIB-4 index<1.3 are at low risk of advanced fibrosis, defined as bridging fibrosis (F3) or compensated fibrosis (F4), and thus can be managed by primary care.^49^,^59^ Patients with an indeterminate or high risk of clinically significant fibrosis (≥F2) or those with discordant results of these noninvasive tests should be referred to a hepatologist for consideration of liver biopsy to guide optimal management. The screening and fibrosis assessment algorithm for MASLD is illustrated in Figure 1. In addition, liver biopsy should be considered in patients whose diagnosis of NASH is uncertain or additional/alternate etiologies are suspected.^60^ position statements 8–10 in Table 1.

## RISK STRATIFICATION AND REFERRAL

### Risk stratification for liver complications

**FIGURE 2:**
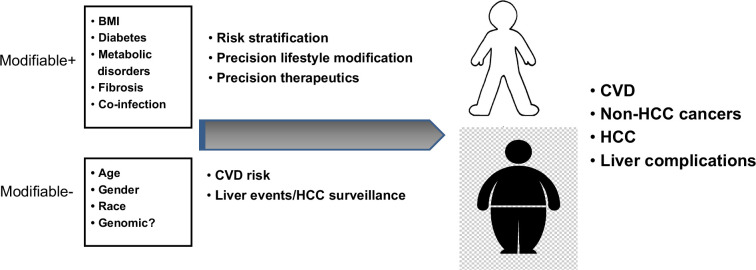
Risk stratification for the patients with metabolic dysfunction–associated steatotic liver disease. Abbreviations: BMI, body mass index; CVD, cardiovascular disease.

The initial step for risk stratification depends on the individualized elucidation of the modifiable and nonmodifiable risks for patients upon diagnosis of MASLD (Figure 2). Fibrosis staging remains to be the initial step for risk stratification in patients with MASLD. Recent practice guidelines recommend that the FIB-4 index be the initial noninvasive test for risk stratification in patients with MASLD based on metabolic risk factors due to its simplicity and ease of use.^49^,^59^,^61^ The surveillance of liver-related complications, such as cirrhosis complications and HCC, should be implemented upon the establishment of advanced fibrosis.

### Risk stratification for CVD and diabetes complications

ASCVD includes coronary heart disease, cerebrovascular disease, and peripheral artery disease.^62^ Key ASCVD risk factors that can be modified are high cholesterol levels, high blood pressure, smoking, diabetes mellitus, and obesity.^63^,^64^ Besides diabetes, MASLD is an independent risk factor for ASCVD.^65^ Therefore, it is recommended that patients with MASLD be referred to a cardiology specialist for further management upon evidence or suspicion of ASCVD.

The long-term risk of developing CKD stage ≥3 is increased 1.45-fold in individuals with MASLD. All risks are independent of age, sex, obesity, hypertension, diabetes, and other conventional CKD risk factors, and the risk appears to increase in parallel with the severity of MASLD.^66^ MASLD carries an increased risk of developing CKD, independent of traditional CKD risk factors. For patients with MASLD who are found to have CKD or albuminuria, it is recommended that they be referred to a nephrology specialist for further management. Liver fibrosis is associated with distal symmetric polyneuropathy. These associations remain significant even after adjusting for BMI, hemoglobin A1c (HbA1c) or plasma glucose, lipid profile, IR, blood pressure, and other distal symmetric polyneuropathy risk factors.^67^,^68^,^69^ Screening patients with MASLD for diabetic microvascular complications is recommended. A causal relationship between MASLD and diabetic microvascular complications remains to be proven.

There is no evidence to support different therapeutic approach between patients with diabetes with or without MASLD. For patients with MASLD at high risk of CVD and diabetes complications, standard management of diabetes to prevent CVD and diabetic complications is recommended.

### When to refer to a hepatologist/diabetologist

The major causes of death in patients with MASLD are CVD, extrahepatic cancers, and hepatic complications. Of note, hepatic fibrosis is associated with the emergence of the major causes of death.^70^ Patients with MASLD with clinical evidence of cirrhosis and advanced fibrosis, assessed by the FIB-4 index or vibration-controlled transient elastography, have a higher incidence of HCC than their counterparts.^71^ The presence of diabetes, male, old age, and a family history of HCC also increase the risk of HCC.^72^ Therefore, diabetes patients should be referred to hepatologists for a complete evaluation of both fibrosis stage and potential existing liver events.

**FIGURE 3:**
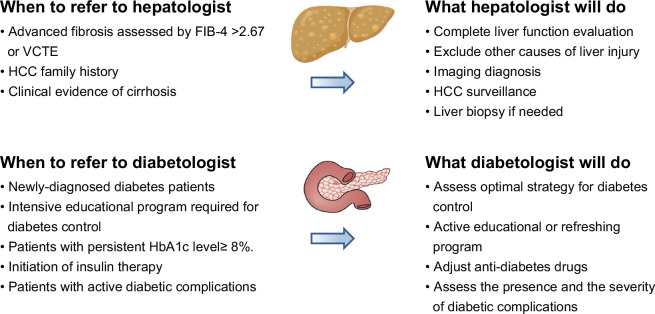
When to refer to a hepatologist/diabetologist in diabetes with metabolic dysfunction–associated steatotic liver disease. Abbreviations: FIB-4, fibrosis-4; HbA1c, hemoglobin A1c; VCTE, vibration-controlled transient elastography.

On the other side, patients with MASLD should be referred to a diabetologist based on existing or potential events. These include patients with newly diagnosed diabetes, intensive educational programs required for diabetes control, patients with poor-controlled diabetes (persistent HbA1c level ≥8%), initiation of insulin therapy, and patients with active diabetic complications (Figure 3). position statements 11–14 in Table 1.

## MANAGEMENT AND PERSPECTIVES

### Prevention of diabetes and MASLD

Lifestyle intervention should be the initial step toward patient care of MASLD. It is intended to lead to weight reduction through a healthy dietary change and increased physical activity tailored to the individual’s tolerance and ability. Lifestyle intervention is also highly effective in preventing or delaying diabetes and improving other cardiometabolic markers.^73^ The 2 major goals of lifestyle intervention for diabetes prevention are to achieve and maintain a minimum of 7% weight reduction and 150-minute moderate-intensity physical activity per week. Despite the lack of a head-to-head comparison, the risk of diabetes development could be reduced by 43% in 7 years, 34% in 10 years, 27% in 15 years, and 39% in 30 years, in different cohort studies.^74^,^75^ The recommended pace of weight reduction for diabetes prevention is 1–2 lb/week, and the reduction of calorie intake by 500–1000 kcal/d is mandatory.^76^ Medical nutritional therapy delivered by registered dietitian nutritionists is associated with a 0.3%–2.0% HbA1c reduction in patients with diabetes.^77^

### Weight reduction

A weight loss of 5% improves pancreatic β-cell function, insulin sensitivity, and hepatic steatosis. However, a more significant weight loss of 7%–10% is necessary for NASH resolution and fibrosis regression. Early evidence was from a randomized controlled trial in a dietitian-led lifestyle modification program or receive usual care for 52 weeks in Hong Kong. It showed that 97% of patients with weight loss >10% had remission of MASLD. Meanwhile, 41% of those with weight loss of 3.0%–4.9% could also achieve the primary outcome of liver fat<5%.^78^

Weight reduction is the most important factor for reducing the risk of incident diabetes. For overweight and obese adults with diabetes, a weight reduction of >5% has a significant effect on metabolic alterations. Weight reduction of ≥10% early in the disease trajectory is associated with a doubling of the likelihood of diabetes remission at 5 years in patients with newly diagnosed diabetes.^79^

### Diet

Dietary sugar consumption is engaged in the phenotypic onset and progression of MASLD. Sugar intake restriction may provide an effective disease prevention and treatment solution.^80^ Carbohydrate intake should emphasize nutrient-dense carbohydrate sources high in fiber (at least 14 g fiber per 1000 kcal) and minimally processed.^81^ Fructose-containing soft drinks are not recommended since they are associated with fibrosis progression in patients with MASLD.^82^,^83^ Omega-3 treatment is beneficial in decreasing liver fat but not for improving NASH or fibrosis.^84^,^85^

The traditional Mediterranean diet may improve glucose metabolism and decrease CVD risk.^81^ Adherence to a traditional Mediterranean diet is inversely associated with the occurrence and severity of MASLD.^86^ The traditional Japanese diet^87^ includes greater intakes of green and yellow vegetables, seaweed/mushrooms/konjac, dairy, fruits, fish, salty, and soybeans/soy products.^88^ There is an inverse relationship between traditional Japanese diet and BMI, triglyceride level, and steatosis. The traditional Japanese diet is also associated with the lower severity of liver fibrosis in patients with MASLD.^89^

In addition to metabolic risks, dietary risks independently drive the global burden of MASLD-related liver mortality. Solid policies to improve the dietary environment for MASLD burden reduction are mandatory for the young generation and the general population.^83^

### Exercise

Physical activity/exercise recommendations are similar for both diabetes patients and diabetes patients with MASLD.^81^ Individualized exercise programs offer greater benefits than standard counseling in adults with diabetes and MAFLD, in particular for those obese.^90^ Structured exercise training elicits an absolute reduction in the intrahepatic triglyceride level that is often proportional to the magnitude of the exercise training and anthropometric improvements.^91^ Exercise is also a fundamental step for diabetes prevention. Achieving at least 150 minutes of physical activity per week decreases diabetes risk by 44%.^92^ In regard to the effect of type of exercise, both aerobic and resistance training improve MAFLD in proportion to treatment engagement and intensity of the program.^93^ The increased physical activity is preferably >150 min/wk of moderate or 75 min/wk of vigorous-intensity physical activity. Both exercise programs, either high or moderate intensity, reduce liver fat and visceral lipids. Patients with MASLD may have unique physiologic limitations to exercise that worsen with fibrosis severity. Therefore, exercise interventions that are personalized and scalable may improve the sustainability of exercise habits in the long term.^94^ For patients with poor cardiopulmonary function, resistance exercise may be more suitable for its low intensity.^90^

### Pharmacotherapeutics for MASLD and diabetes

The first antidiabetic drug for NASH treatment was peroxisome proliferator-activated receptor gamma agonist. Pioglitazone, a proliferator-activated receptor gamma agonist, has been shown to improve IR, primarily targeting adipose tissue and improving lipid storage/redistribution and glucose utilization.^95^ A recent first‑in‑Asian double-blind, randomized, placebo-controlled trial demonstrated that a 24-week pioglitazone treatment was well tolerated and effective in improving liver histology and reducing liver steatosis in Asian patients with NASH. The patients receiving pioglitazone had significant NASH improvement without worsening fibrosis, decreasing liver fat on MRI-derived proton density fat fraction, decreasing lipid profile, and normalizing liver enzymes.^96^

Glucagon-like peptide 1 receptor agonists (GLP-1 RAs) have beneficial effects on ASCVD and weight loss. GLP-1 RAs normalize aminotransferase levels, decrease HbA1c level, reduce liver fat content, and improve histological manifestations in patients with MASLD.^97^,^98^,^99^ Recent post hoc analysis of data from a phase II study demonstrated that a significant treatment efficacy of GLP-1 RAs was observed in patients with NASH in terms of steatosis, necroinflammation, and fibrosis in continuous scores.^100^

Sodium-glucose cotransporter 2 inhibitors reduce lower blood glucose levels, cause modest weight loss, and are associated with robust cardiorenal benefits. SGLT-2i may have favorable effects on liver fat content and liver enzymes in diabetes patients with MASLD.^97^ SGLT-2i has favorable long-term histological and clinical impacts for patients with MASLD and diabetes.^101^,^102^ There are still many antidiabetes drugs with potential benefits for fatty liver disease, which are summarized in Table 2.

**TABLE 2:** Summary of efficacy and adverse effects of antidiabetic drugs for patients with MASLD and diabetes

	Glycemic control	Body weight reduction	Atherosclerotic cardiovascular disease	Heart failure	Diabetic kidney disease	Hypoglycemia risk	Side effects	Outcomes
Glucagon-like peptide-1 receptor agonist	High	High	Beneficial^a^	No significant effect	Beneficial in albuminuria improvement^a^	Low	Gastrointestinal adverse effects, including nausea, anorexia, vomiting, potential pancreatitis	Transaminase level: decreased Liver steatosis: decreased Disease activity: improved Fibrosis: decreased
Glucagon-like peptide-1 receptor agonist/glucose-dependent insulinotropic polypeptide	High	High	Lack of evidence	Lack of evidence	Lack of evidence	Low	Gastrointestinal adverse effects, including nausea, anorexia, and diarrhea	Transaminase level: decreased Liver steatosis: decreased Disease activity: improved Fibrosis: decreased
Sodium-glucose cotransporter 2 inhibitor	Intermediate	Intermediate	Beneficial^b^	Beneficial^b^	Beneficial^b^	Low	Urogenital tract infection, diabetic ketoacidosis, volume depletion	Transaminase level: decreased Liver steatosis: decreased Disease activity: no significant effect
Metformin	High	Intermediate	Potentially beneficial	No significant effect	No significant effect	Low	Gastrointestinal adverse effects, potential lactic acidosis	Transaminase level: decreased/unchanged Liver steatosis: unchanged Disease activity: no significant effect
Thiazolidinediones	High	Potentially weight gain	Potentially beneficial	Increased risk	Lack of evidence	Low	Edema, exacerbated heart failure	Transaminase level: decreased Liver steatosis: decreased Disease activity: improved
Dipeptidyl peptidase IV inhibitor	Intermediate	No significant effect	No significant effect	No significant effect	Beneficial in improvement of albuminuria (linagliptin)	Low	Joint pain, bullous pemphigous, pancreatitis	Transaminase level: decreased/unchanged Liver steatosis: unchanged Disease activity: no significant effect
Sulfonylurea/glinides	Intermediate/high	Weight gain	No significant effect	No significant effect	No significant effect	Yes	Hypoglycemia	Lack of evidence
Insulin	High	Weight gain	No significant effect	No significant effect	No significant effect	Yes	Hypoglycemia	Transaminase level: decreased/unchanged Liver steatosis: unchanged Disease activity: no significant effect
α-glucosidase inhibitor	Intermediate	Low to intermediate	No significant effect	Lack of evidence	Lack of evidence	Low	Gastrointestinal	Lack of evidence

^a^ Liraglutide, semaglutide, dulaglutide.

^b^ Empagliflozin, dapagliflozin, canagliflozin.

### Nonpharmacological interventions

Endoscopic bariatric and metabolic therapies include procedures that require flexible endoscopy for weight loss or treatment of glucose intolerance. Intragastric balloon, which occupies space in the stomach, is currently the most commonly used endoscopic bariatric and metabolic therapies; however, it should be removed 6 months after placement. In addition to a significant decrease of transaminase and fasting plasma glucose levels, the weight reduction reached 12.0% at the time of intragastric balloon removal in morbidly obese Taiwanese patients.^103^ Metabolic surgery and metabolic endoscopic techniques significantly lead to effective body weight loss and resolution of histological changes of steatosis, inflammation, and fibrosis.^104^ However, they are costly and limited by the potential acute and/or chronic postoperative complications.

### Patients with other liver diseases

Patients with viral hepatitis, including chronic hepatitis B, CHC, and ALD, would compromise the liver disease severity, and are at a high risk of liver complications, including liver cirrhosis, HCC, and liver-related mortality in patients with MASLD.

For patients with MASLD with concurrent viral hepatitis, the essential step to reduce the risk of liver-related complications is suppressing HBV activity and eradicating HCV. Absolute alcohol abstinence is particularly recommended for patients with liver cirrhosis.^105^ position statements 15–17 in Table 1.

## PERSPECTIVES

### Clinical trials

Maintenance of lifestyle modification to alleviate or to reverse MASLD/NASH is a challenging task. In 2024, Food and Drug Administration approved the first drug, a thyroid hormone receptor beta–selective agonist, resmetiron, for the treatment of NASH with F2–3 liver fibrosis.^106^ Meanwhile, >20 drugs are being investigated.^104^

In consideration of the disease heterogenicity of MASLD, precision medicine with the assistance of novel technology such as artificial intelligence, machine learning, etc., to identify high-risk genotypes and to give more appropriate treatment regimens will be more effective and efficient.^107^,^108^

Despite of impressive weight control (10%–30% in 1 y) and regression of liver steatosis, the durability of GLP-1RA in weight control is limited and has to be combined with lifestyle change. The goal of reducing liver fibrosis has to be carefully examined in future trials. Moreover, whether long-term therapies can prolong healthy, overall survival of people with MASLD, especially those with significant liver fibrosis, still awaits the outcomes from large-scale and longer clinical trials. Combination therapies would be a potential strategy to provide promising results in the direction.

Asian people are more prone to develop MASLD and lean MASLD than other races in the same BMI level. Therefore, results derived from clinical trials conducted in the western may not be generalized to the Asian people without additional verification. It is also worth noting that viral hepatitis is common in Asia. Clinical trials conducted in patients with MASLD without viral hepatitis may not be extrapolated to patients with viral hepatitis.

The increasing incidence of obesity in the young is in parallel increase in MASLD.^109^ It suggests a potential public health challenge in the near future and should be deemed as an urgent issue. Clinical trials targeting the younger generation are urgently needed.

### HCC prevention

Patients with MASLD with advanced fibrosis are at high risk of HCC, irrespective of cirrhosis. The risk increases with presence of diabetes, obesity, and metabolic syndrome.^1^ Inadequate diabetes control and even prediabetes are consistently associated with a higher risk of HCC, and long-term diabetes control should be considered in comprehensive cirrhosis care.^110^,^111^ Metformin, statins, coffee, and aspirin have been investigated for HCC prevention for various etiologies of liver diseases.^35^ Metformin promotes modest weight loss, ameliorates hyperinsulinemia, and potentially prevents HCC development.^112^ In patients with MASLD with diabetes, metformin use had a 20% risk reduction of HCC.^33^ There was no evidence of fibrosis improvement among patients receiving pioglitazone or statins. position statements 18 in Table 1.

## SUMMARY

**FIGURE 4:**
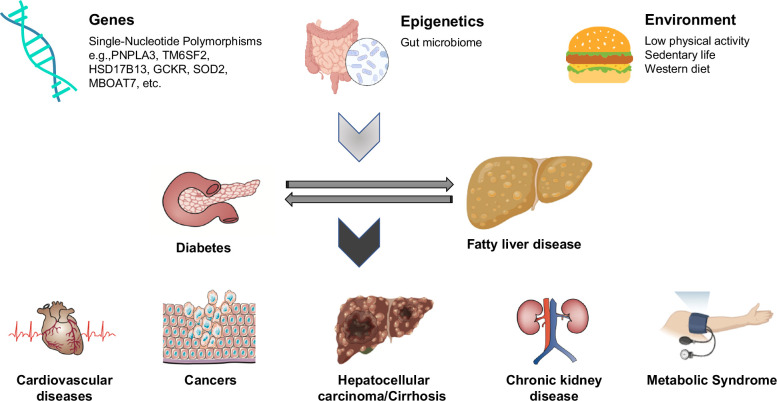
The spectrum and related outcomes of metabolic dysfunction–associated steatotic liver disease.

**FIGURE 5:**
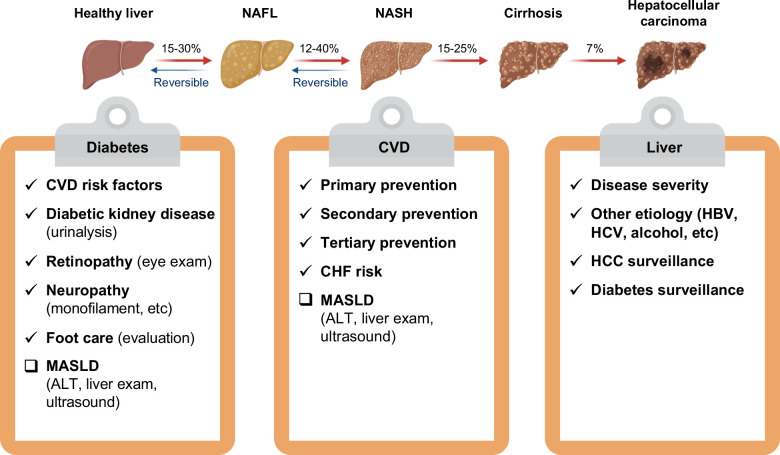
The comprehensive directions of patient care in MASLD. Abbreviations: CHF, congestive heart failure; CVD, cardiovascular disease; MASLD, metabolic dysfunction–associated steatotic liver disease.

The prevalence and incidence of SLD have been rapidly progressing in the past several decades throughout the Asia-Pacific region in parallel with the rapid westernization of the region. Despite a significantly lower BMI and lower rates of obesity compared to other ethnic groups, Asians have a significant prevalence of MASLD and other metabolic disorders. The interaction and impacts are bidirectional between MASLD and diabetes in terms of disease course, risk, and prognosis. The extensive investigation of metabolic liver diseases with complex mechanisms is essential for diagnosis, management, and outcome prediction. During the disease course of MASLD, liver disease severity assessment is mandatory for liver-related outcome prediction. Meanwhile, patient care, according to current diabetes and CVD guidelines, is a fundamental effort in a clinical setting. The efforts will provide more clues for the elucidation of genetic, epigenetic, and environmental risk factors through the occurrence of hepatic and extrahepatic events (Figure 4). Meanwhile, there is an urgent need for the early detection and management of metabolic liver disorders due to the heterogenicity and complexity of the underlying mechanisms as well as the global surge in prevalence. A multidisciplinary approach with lifestyle modification such as weight reduction, exercise, dietary treatment, restriction of alcohol, and abstinence from smoking should always be encouraged (Figure 5). The first position statement of MASLD in diabetes was constructed by enormous and enthusiastic efforts by the major academic societies of hepatology and diabetology in Taiwan. Hopefully, the collaborative efforts and statements listed will provide practical patient guidance for primary care professionals.
